# Natural Killer Cell Tolerance Persists Despite Significant Reduction of Self MHC Class I on Normal Target Cells in Mice

**DOI:** 10.1371/journal.pone.0013174

**Published:** 2010-10-04

**Authors:** Petter Brodin, Tadepally Lakshmikanth, Ramit Mehr, Maria H. Johansson, Adil Doganay Duru, Adnane Achour, Mali Salmon-Divon, Klas Kärre, Petter Höglund, Sofia Johansson

**Affiliations:** 1 Department of Microbiology, Tumor and Cell Biology, Karolinska Institutet, Stockholm, Sweden; 2 The Mina and Everard Goodman Faculty of Life Sciences, Bar-Ilan University, Ramat-Gan, Israel; 3 Department of Medicine, Center for Infectious Medicine, Karolinska University Hospital Huddinge, Karolinska Institutet, Sweden; 4 Department of Applied Physics, Experimental Biomolecular Physics, Royal Institute of Technology, AlbaNova University Center, Stockholm, Sweden; 5 European Molecular Biology Laboratory (EMBL) European Bioinformatics Institute, Wellcome Trust Genome Campus, Cambridge, United Kingdom; Centre de Recherche Public de la Santé, Luxembourg

## Abstract

**Background:**

A major group of murine inhibitory receptors on Natural Killer (NK) cells belong to the Ly49 receptor family and recognize MHC class I molecules. Infected or transformed target cells frequently downmodulate MHC class I molecules and can thus avoid CD8^+^ T cell attack, but may at the same time develop NK cell sensitivity, due to failure to express inhibitory ligands for Ly49 receptors. The extent of MHC class I downregulation needed on normal cells to trigger NK cell effector functions is not known.

**Methodology/Principal Findings:**

In this study, we show that cells expressing MHC class I to levels well below half of the host level are tolerated in an *in vivo* assay in mice. Hemizygous expression (expression from only one allele) of MHC class I was sufficient to induce Ly49 receptor downmodulation on NK cells to a similar degree as homozygous expression, despite a strongly reduced cell surface level of MHC class I. Co-expression of weaker MHC class I ligands in the host did not have any further effect on the degree of Ly49 downmodulation. Furthermore, a single MHC class I allele could downmodulate up to three Ly49 receptors on individual NK cells. Only when NK cells simultaneously expressed several Ly49 receptors and hemizygous MHC class I levels, a putative threshold for Ly49 downmodulation was reached.

**Conclusion:**

Collectively, our findings suggest that in interactions between NK cells and normal untransformed cells, MHC class I molecules are in most cases expressed in excess compared to what is functionally needed to ensure self tolerance and to induce maximal Ly49 downmodulation. We speculate that the reason for this is to maintain a safety margin for otherwise normal, autologous cells over a range of MHC class I expression levels, in order to ensure robustness in NK cell tolerance.

## Introduction

Natural Killer cells are important in the defense against tumor cells and virally infected cells. Their killing activity is regulated by a balance between several activating and inhibitory receptors [1]. The activating receptors bind a diverse array of ligands, some of which are stress-induced or virally encoded [2]. Many inhibitory receptors expressed on NK cells recognize MHC class I molecules, which are expressed on the majority of healthy cells. In case MHC class I molecules are lost, NK cell killing may be induced. This response is termed “missing-self” recognition [3], and may operate as a defense against certain virus-infected and neoplastic cells on which MHC class I is downmodulated in order to avoid CD8^+^ T cell recognition. Thus, a target cell can become NK cell sensitive either by upregulation of ligands for activating NK cell receptors or by downregulation of MHC class I [4], [5], [6]. These mechanisms can operate independently but may also synergize to enhance NK cell sensitivity [7].

The recognition of self MHC class I in the murine system depends largely on Ly49 receptors. These receptors constitute a family with both activating and inhibitory members, where the inhibitory receptors have MHC class I as ligands [8]. A specific interaction between an MHC class I molecule and an inhibitory Ly49 receptor leads to transmission of a signal through the Immunoreceptor Tyrosine-based Inhibitory Motif (ITIM), situated in the cytoplasmic domain of Ly49 receptors. ITIM phosphorylation leads to recruitment of the phosphatase Shp-1, whose activation leads to dephosphorylation of substrates in the downstream propagation of activating signals [9].

The Ly49 receptor family contains several members with distinct MHC class I-binding patterns. NK cells with full functionality only mature if correct matching between Ly49 receptors with self MHC class I occur; presumably a mechanism to ensure self tolerance [10], [11]. Since the Ly49 and MHC class I loci are genetically unlinked, an educational process is needed to distinguish and promote NK cells with self MHC specificity [12], [13], [14]. The mechanistic details of this process are poorly understood. What is clear, however, is that there is a direct role for MHC class I alleles in this process. Different MHC class I alleles educate NK cells in a quantitatively different manner, i.e. they have different educating impact on NK cells [15], [16]. We have recently shown that the self MHC class I alleles that are present in an individual dictate the magnitude of response of individual NK cells and control the quality of effector responses that each NK cell performs upon stimulation. To explain this influence, we have proposed a “rheostat model” of NK cell education [17], [18].

Apart from interacting with MHC class I on other cells, Ly49 receptors also interact with MHC class I in their own cell membrane, i.e. in *cis* [19]. By means of *cis* interaction, self MHC class I molecules sequester Ly49 receptors, preventing them from reaching the NK - target cell synapse [20]. As a consequence, inhibition is reduced and the threshold for activation is lowered.

When Ly49 receptors interact with MHC class I, their cell surface levels are reduced. There are two reasons for this. Firstly, receptors are internalized upon MHC class I ligand-binding in *trans*, and secondly, Ly49 - MHC *cis* interactions masks the antibody binding epitopes on the Ly49 receptors, which appears as a lower detected expression level [19], [21], [22], [23]. Regardless of the underlying mechanism, this decrease in detected Ly49 receptor level represents an indirect sign of a specific Ly49-MHC class I interaction, and, as such, is useful for studies of Ly49 receptor specificity [19], [21], [22], [24], [25], [26]. We will from hereon refer to this decrease in detected receptor expression level, as receptor downmodulation.

The findings mentioned above suggest that Ly49 interactions with different MHC class I *alleles* are interpreted in a quantitative way by the NK cell [16], [17]. Another interesting question is whether the MHC class I expression *level* of a given allele impose a quantitative influence on Ly49 receptor signaling, especially in interactions with normal autologous cells. Most studies attempting to establish the role of MHC class I levels for NK cell inhibition used transformed target cells with different MHC class I levels. These studies came to the conclusions that the transition from protection to loss of protection is not controlled continuously over a range of MHC class I expression levels, but by a threshold effect [13], [27], [28]. A recent paper showed that hemizygous expression of MHC class I did not trigger killing by NK cells from MHC homozygous mice, despite a significant reduction in expression of MHC class I on the hemizygous target cells [29]. These results, and the results from previous studies using tumor cell targets [13], [27], [28], suggest that NK cells can tolerate a large reduction in self MHC class I before they are triggered *in vitro*.

Whether NK cell responses *in vivo* are subject to a similar regulation is not known. If they are, it is of great importance to identify how much self MHC class I must be lost before NK cell rejection of self cells occur *in vivo*. In the present paper, we have studied the overall *in vivo* rejection of primary splenocytes expressing different levels of surface MHC class I, aiming to elucidate how much MHC class I is needed on the normal healthy target cell to protect it from NK cell killing. We found that normal, resting cells were tolerated by NK cells over a wide range of MHC class I cell surface levels, rejection ocurring only when MHC class I levels fell below approximately 20% of the host levels, supporting a model in which a larger number of MHC class I molecules than inhibitory receptors are present *in vivo*. Further supporting this notion, a reduction of endogenous MHC class I levels down to around half of the level in MHC homozygous mice still led to full downmodulation of a number of investigated Ly49 receptors. In addition, coexpression of several Ly49 receptors recognizing the same MHC class I allele on individual NK cells did not reveal any competition between different Ly49 receptors for MHC class I ligands. Altogether, our results suggest that MHC class I molecules are expressed in excess compared to what is needed for maximal downmodulation of Ly49 receptors. We speculate that there is a large margin in the amount of expressed MHC class I needed to protect normal cells from being killed by NK cells, which may reflect a need to secure self tolerance over a large range of natural variations in MHC class I expression.

## Materials and Methods

### Mice

Mice were bred and maintained at the Department of Microbiology and Tumor and Cell Biology (MTC, Karolinska Institutet, Stockholm, Sweden). C57BL/6 (abbreviated B6, H-2K^b^D^b^) and B6.Tap^−/−^ mice were originally obtained from the Bomholt Gaard breeding and research center (Ry, Denmark). D8 mice (transgenic for H-2D^d^ on B6 background) and L3 mice (transgenic for H-2L^d^ on B6 background) have been previously described [15], [30]. B6.K^b−/−^, B6.D^b−/−^ and B6.K^b−/−^D^b−/−^ mice were generated as described [31], [32], [33]. Mice expressing H-2D^d^ (B6.K^b−/−^D^b−/−^D^d+/+^) or H-2L^d^ (B6.K^b−/−^D^b−/−^L^d+/+^) alone on B6 background were generated as described [16]. For simplicity, the MHC class I molecules will be denoted by their allelic (e.g. D^b^, L^d^) names only throughout the paper from hereon, and mice will be denoted by the MHC alleles they do express. See Table 1 for designations and full genotypes.

**Table 1:** Genotypes of the mice used in this study.

Designation in this study	Genotype
MHC^−/−^	B6.K^b−/−^D^b−/−^
*Homozygote mice*	
K^b^D^b+/+^	B6
K^b+/+^	B6.D^b−/−^
D^d+/+^	B6.K^b−/−^D^b−/−^D^d+/+^
*Hemizygote mice*	
K^b^D^b+/−^	B6.K^b+/−^D^b+/−^
K^b+/−^	B6.K^b+/−^D^b−/−^
D^d+/−^	B6.K^b−/−^D^b−/−^D^d+/−^
*Multiple MHC mice*	
K^b^L^d^	B6.D^b−/−^L^d+^
K^b^D^d^	B6.D^b−/−^D^d+^
D^b^L^d^	B6.K^b−/−^L^d+^
D^b^D^d^	B6.D^b−/−^D^d+^
K^b^D^b^L^d^	B6.L^d+^
K^b^D^b^D^d^	B6.D^d+^

**Figure 1:**
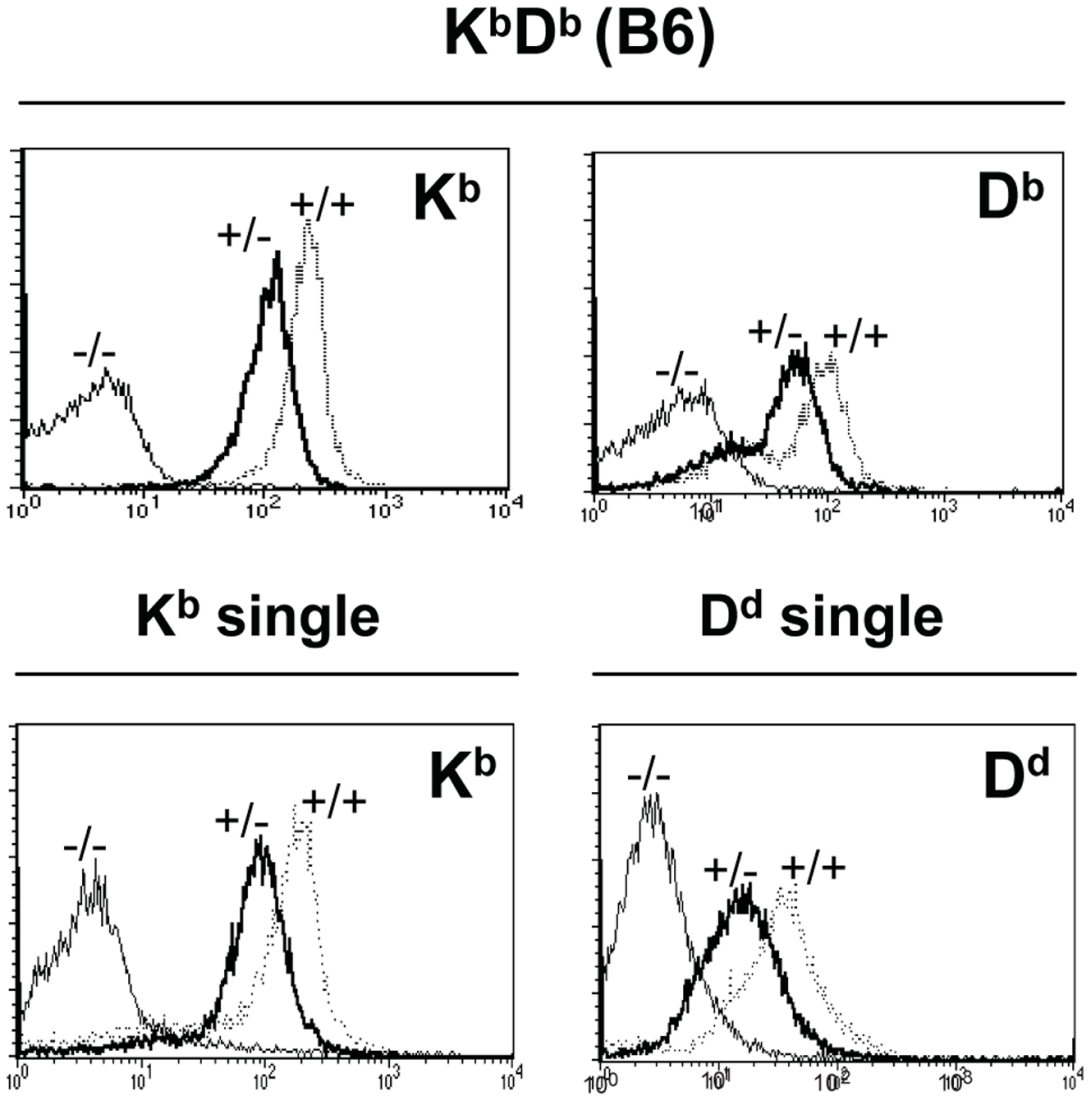
MHC class I expression in mice hemizygous or homozygous for MHC class I. Expression levels of MHC class I molecules in K^b^D^b^ mice (upper two panels), K^b^-single mice and D^d^-single mice (lower two panels), as measured by flow cytometry. Thick black, thin grey and intermediate grey lines represent expression in hemizygous, homozygous and MHC class I-deficient mice, respectively. Figures show one representative mouse out of 14 stained for each mouse strain.

Mice hemizygous for both K^b^ and D^b^ (with one allele at each locus expressing an MHC molecule, and the other being null, K^b+/−^D^b+/−^), were generated by crossing B6 mice with B6.K^b−/−^D^b−/−^ mice. Mice hemizygous for either K^b^ or D^d^ alone was also generated by crossing K^b+/+^ or D^d+/+^ mice, respectively, with B6.K^b−/−^D^b−/−^ mice. The MHC class I cell surface expression in these hemizygous mice, relative to the homozygotes, is shown in Figure 1.

**Figure 2:**
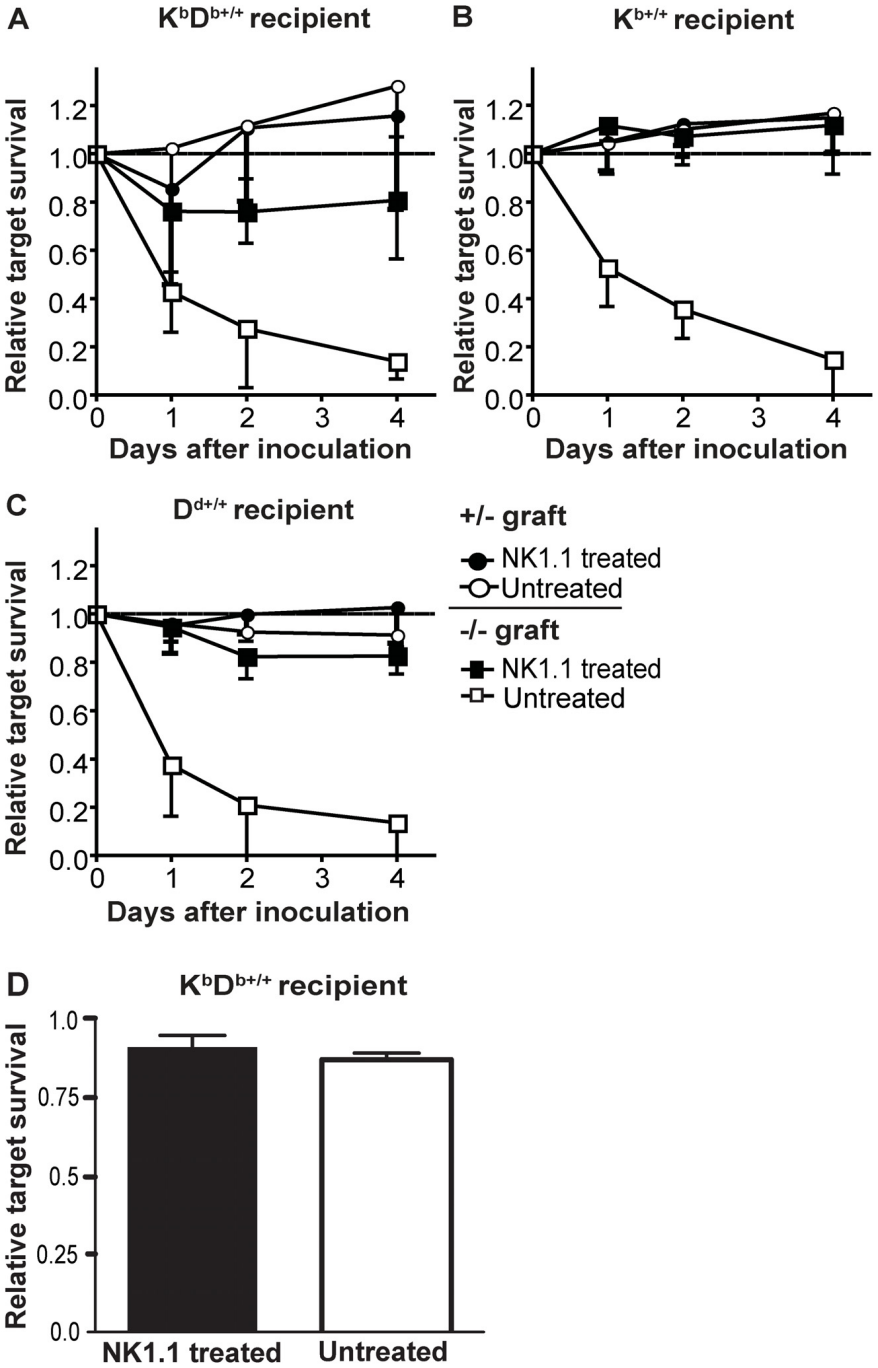
MHC class I homozygous mice fail to kill MHC hemizygous splenocytes *in vivo*. **A–C**) Inoculation of CFSE^high^ labeled MHC class I hemizygous (white circles) or MHC class I-deficient (white squares) splenocytes as targets, and CFSE^low^ syngeneic controls, into the indicated MHC class I homozygous recipient. Black symbols represent the corresponding recipient depleted of NK cells. **D**) Over-night rejection of CFSE labeled K^b+/−^D^b+/−^ targets compared to syngeneic controls in tilorone treated, NK1.1 depleted or untreated K^b+/+^D^b+/+^ recipients. Graphs (A–C) show mean values and standard deviations from 2–3 experiments with total n = 10 (K^b^D^b^) and n = 4 mice (K^b^ and D^d^), while in D, data show one representative out of 2 experiments performed (n = 2 mice per group).

Mice expressing single MHC class I alleles (homozygously) were crossed to generate F_1_ generations with the following genotypes: K^b^+D^d^, D^b^+D^d^, K^b^+L^d^ and D^b^+L^d^ [16]. It should be noted that the breedings of double and triple MHC class I mice used in the experiments for modeling of Ly49 downmodulation were set up as alternatively hemizygote or homozygote of different MHC class I alleles, and that consequently the expression levels of K^b^, D^b^ and D^d^ differed between the mice (Table S1). Interestingly, the expression levels of L^d^ did not follow the gene dosage pattern demonstrated by other alleles. However, as proposed by the experiments shown in Figure 2, the lower expression levels of MHC class I in hemizygous mice did not have any effect on Ly49 down-modulation, when compared to homozygous expression (Figure S1).

### CFSE labeling and in vivo killing of CFSE-labeled cells

**Figure 3:**
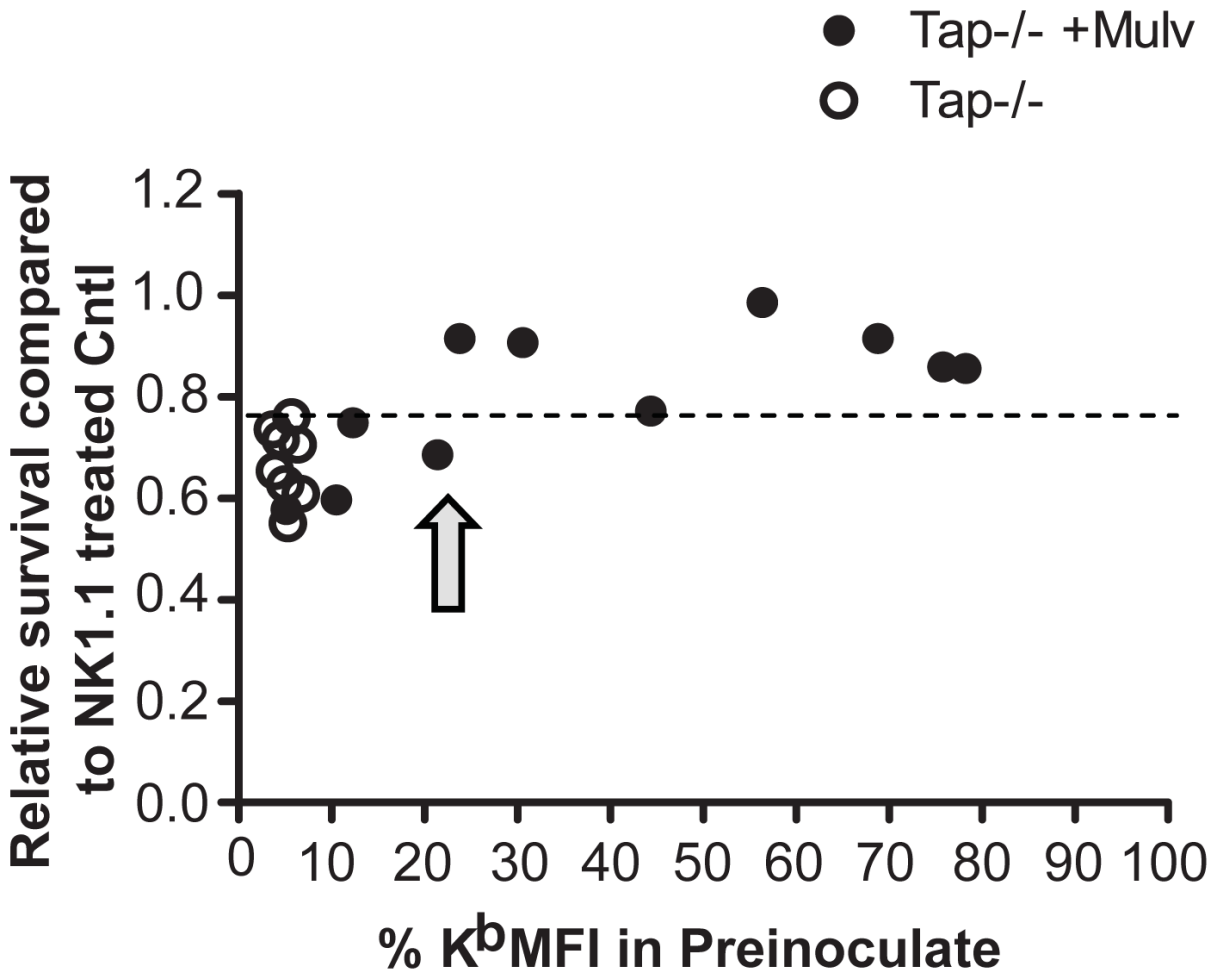
*In vivo* NK cell rejection of cells with peptide-stabilized MHC class I expression. K^b^ single mice were injected with CFSE^high^ B6.Tap^−/−^ cells unpulsed or pulsed with Mulv peptide, mixed with CFSE^low^ syngeneic control cells. The cells were stained for K^b^ at the time of inoculation, and the K^b^ MFI level in % of the syngeneic controls was calculated. These values are indicated on the x axis. The rejection was assessed in the spleen after 8 hrs, and is shown on the Y axis. CFSE ratio between target cells and syngeneic controls was calculated relative to the NK1.1 treated group in each experiment, to compensate for inter-experimental variation. Each dot represents the mean of 2 mice/group in each experiment, with total n = 32 mice for the Tap+Mulv groups and n = 23 mice for unpulsed Tap^−/−^ groups (NK depleted and non-depleted). The dashed line indicates the minimal amount of killing of unpulsed cells that occurred in any experiment. Data in the graph were obtained from a total of 8 experiments. The difference between rejection of unpulsed and Mulv pulsed cells was statistically significant (P = 0.0018 for all experiments with an induced K^b^ expression of at least 23% of control, compared to NK1.1 controls in the same experiments).

The method for measuring *in vivo* NK cell mediated rejection by CFSE labeling of the target cells has been previously described [6]. It is a quantitative method performed with resting, non-proliferating targets and non-irradiated recipients. In brief, spleen cells from syngeneic and test mice were stained with 0.3 and 3 µM, respectively, of the dye 5,6-carboxyfluorescein diacetate succinimidyl ester (CFSE; Molecular Probes). 10^7^ cells of each type (syngeneic control and test population) were mixed and co-injected intravenously (i.v.) into the same recipients. After 18–24 hours (8 hours in Figure 3), the spleens were taken out, single cell suspensions were made and the relative number of cells in each CFSE population was measured either by a FACScan or a LSRII (BD Biosciences, Mountain View, CA, USA). In some experiments blood was taken out from the tail vein every 2 hrs to analyze the stability of the Mulv-stabilized B6.Tap^−/−^ cells injected i.v. The relative survival of CFSE^high^ cells compared to CFSE^low^ cells, providing a quantitative estimate of missing self rejection, was calculated as follows: (acquired number of CFSE^high^ cells in sample/acquired number of CFSE^low^ cells in sample)/(acquired number of CFSE^high^ cells in injection mix/acquired number of CFSE^low^ cells in injection mix). At least 2000 control (CFSE^low^) cells were acquired for each sample. When stated, recipient mice were given 2 mg tilorone analogue (T-8014 or T-7514; Sigma-Aldrich, Stockholm, Sweden) orally one day before injection of donor cells. Tilorone is an interferon-inducer, which in non-irradiated recipients has similar effects to Poly I:C (our observations and [34]). For NK cell depletions, 200 µg PK136 (anti-NK1.1, mouse IgG2a) or TMβ-1 (anti-IL-2Rβ, rat IgG2b) were given intraperitoneally two days before the experiment. PK136 and TMβ-1 were purified from hybridoma supernatants by MabTech (Stockholm, Sweden). These two antibodies have a similar NK depleting efficiency (our own unpublished observation).

### Peptide-induced stabilization of K^b^ on B6.Tap^−/−^ target cells inoculated in vivo

B6.Tap^−/−^ splenocytes isolated and treated aseptically were counted and pulsed over-night with 0.2–10 µM Moloney murine leukemia virus (Mulv) peptide (SSWDFITV [35]) at 26°C in RPMI medium containing 2% Fetal Calf Serum. The stability of K^b^ on the Mulv pulsed cells *in vivo* was tested in NK1.1 treated K^b^ single mice (Figure S2A). In this representative experiment, the K^b^ levels expressed by the Mulv pulsed B6.Tap^−/−^ cells in the pre-inoculate were 64% and dropped to 32% within 2 hours at 37°C, relative to K^b^-syngeneic splenocytes. The K^b^ levels were thereafter maintained up to the end of the experiment, 6–8 hrs after inoculation. For rejection experiments, Mulv-pulsed target cells were labeled with 3 µM CFSE and injected intravenously into K^b^-single mice, together with a K^b^-single control cell population labeled with 0.6 µM CFSE. After 8 hrs, the spleens were taken out and the ratio between these two populations was calculated as described above.

### Antibodies and FACS analysis

Splenocytes were isolated and NK cells were enriched by nylon wool separation. Fc receptors were blocked by incubation with 2.4G2 (anti-FcγRIII). Anti-NK1.1 (PK136) -FITC, -PE and -PerCP, anti-CD3ε-PerCP (145-2c11), PE-Cy5, anti-CD19 PerCP-Cy5.5 (1D3), anti-Ly49G2-APC (4D11), anti-Ly49I-PE (YLI-90), anti-Ly49F-PE (HBF-719), anti-H-2K^b^-FITC and -PE, Anti-H-2K^b^-biotin, anti-H-2D^b^-FITC, and streptavidin-PE were purchased from BD Biosciences (Stockholm, Sweden). Anti-Ly49A (YE1/48) hybridoma was grown in our laboratory, protein G purified and subsequently conjugated to Alexa633 (Molecular Probes). Anti-Ly49C-biotin and hybridoma (4LO3311) were a kind gift from Suzanne Lemieux. 4LO3311 supernatant was used and secondary stained using an anti-mouse IgG3 PE-Cy7 (Southern Biotech). Anti-NK1.1 APC-Cy7 (PK136) and anti-Ly49A biotin (YE1/48) were purchased from Biolegend. Conjugation kit QD605, Pacific Blue, the dead cell marker Vivid Aqua and streptavidin QD565 were purchased from Invitrogen. Flow cytometry was performed on a FACScan, FACSAria or an LSRII Special Order System (BD Biosciences, Mountain View, CA, USA) and analyses were made using the FlowJo (ThreeStar, CA) or Cellquest softwares. For analysis of receptor expression, cells were gated on lymphocytes (Forward Scatter/Side scatter) and on NK1.1^+^CD3ε^−^ cells. For analysis of Ly49 receptor expression levels in NK cell subsets, conjugates and dead cells were excluded (FSC-A/FSC-H) and Vivid^+^ respectively, followed by NK identification by CD19^−^CD3ε^−^, NK1.1^+^ gating. Exclusion of Ly49D, NKG2A and Ly49I^+^ cells were done and analysis of Ly49A, -C and -G2 expressing subsets were made.

### Downmodulation index

The Ly49 expression levels in single MHC class I and MHC^−/−^ mice have been published [16]. The relative expression levels *(E)* of Ly49 receptors in MHC class I-expressing mice compared to MHC^−/−^ mice were calculated from the fluorescence intensity *(FI)* as: E = 100 * (median FI)/(median FI in MHC ^−/−^ *mice)*. A down-modulation index (DMI) for each receptor was calculated from E as: *DMI = 100−E*.

### Calculation of expected down-regulation values under the additive, dominant, or average model

For mice expressing a combination of two or three MHC class I molecules, we calculated the expected values using three different models. In all these models, the DMI for each MHC molecule was taken from the DMI of the respective single MHC mouse expressing that molecule. The models were as follows: 1) The expected value of DMI if the effects of different MHC molecules were additive, defined for each mouse as: *DMI (additive)  =  SUM of DMIs of all MHC molecules in this mouse*. For instance, the expected DMI *(additive)* for Ly49C in K^b^D^b^ mice was calculated as DMI in D^b^-single mice + DMI in K^b^-single mice. 2) The expected value of DMI if the effects of different MHC molecules were averaged, defined for each mouse as: *DMI (average)  =  AVERAGE of DMIs of all MHC molecules in this mouse*. 3) The expected value of DMI if binding to the most downmodulating MHC class I dominates, i.e. for each mouse: *DMI (dominant)  =  MAX of DMIs of all MHC molecules in this mouse*. For instance, the expected DMI for Ly49C in K^b^D^b^ mice under the dominance model would equal the DMI in K^b^ single mice.

### Statistical analysis

**Figure 4:**
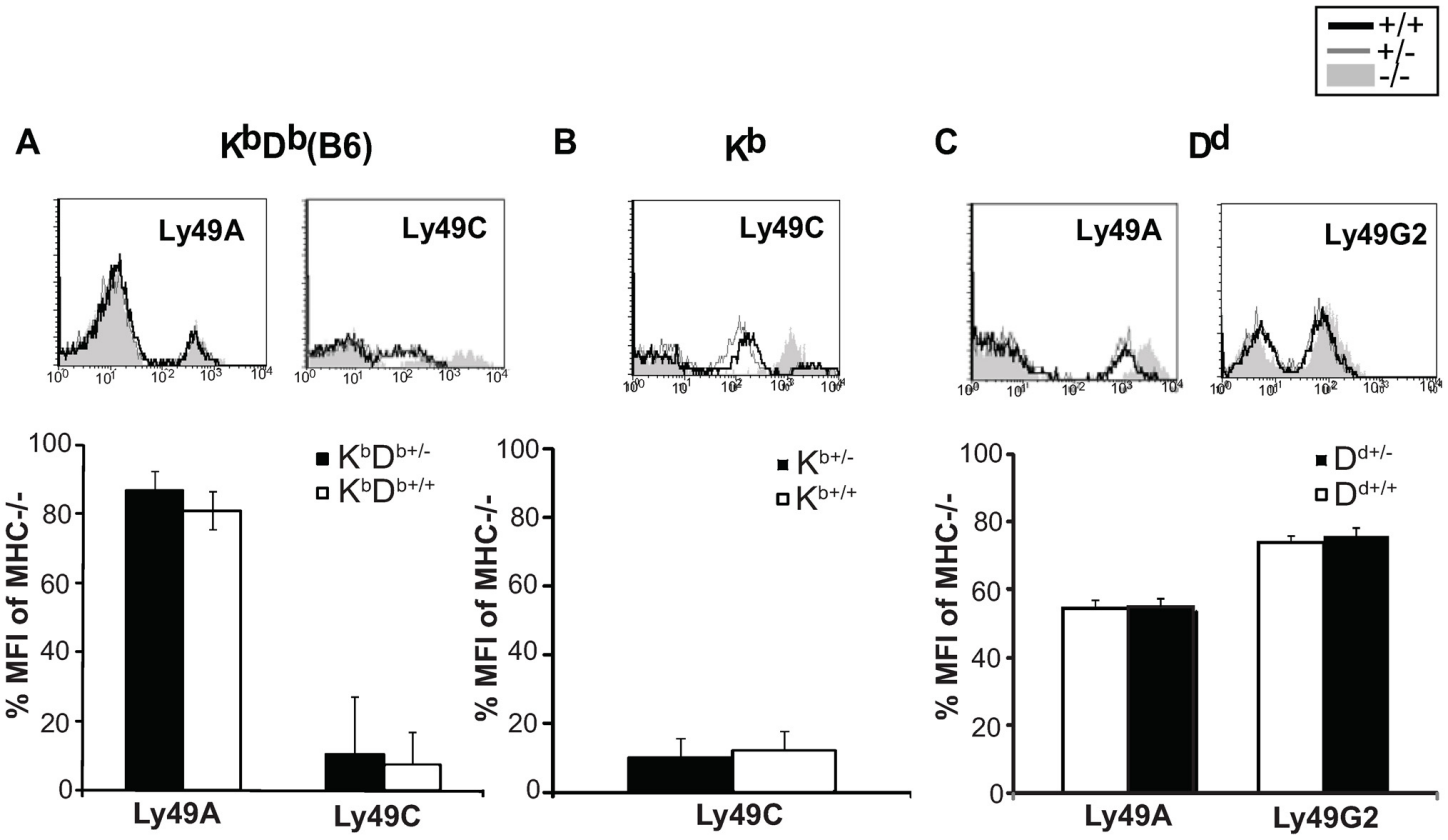
Ly49 expression levels in MHC class I hemizygote and homozygote mice. Ly49 expression on NK1.1^+^CD3^−^ cells in **A**) K^b^D^b^, **B**) K^b^ and **C**) D^d^ mice. Filled grey histograms represent expression in MHC class I-deficient mice, black thick line expression in homozygous mice and grey line in hemizygous mice. Data represents one out of two experiments performed. The bar graphs show expression level of Ly49A, -C, or –G2 presented as % of median fluorescence intensity compared to MHC^−/−^ mice, gated on the positive population for each receptor. Data are the means of a total of 4 mice in each group. Error bars show standard deviation.

**Figure 5:**
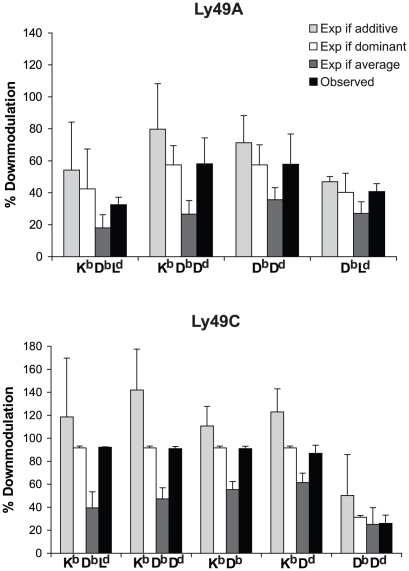
Modeling of the effect of having multiple MHC class I ligands on Ly49 downmodulation. Degree of Ly49A and -C downmodulation in mice expressing more than one MHC class I, and different models for their combined downmodulating effect. The “observed” levels were measured on freshly isolated NK1.1^+^ CD3^−^ splenocytes. The expression is shown as Downmodulation Index (DMI), which means % reduction of median fluorescence intensity compared to the MHC^−/−^ mouse. Values are averages of 3–7 experiments. The modeled values show expected DMI if the downmodulating effect of the MHC class I molecules were additive, dominant, or averaged. See Material and Methods for calculations. Error bars show standard deviations.

**Figure 6:**
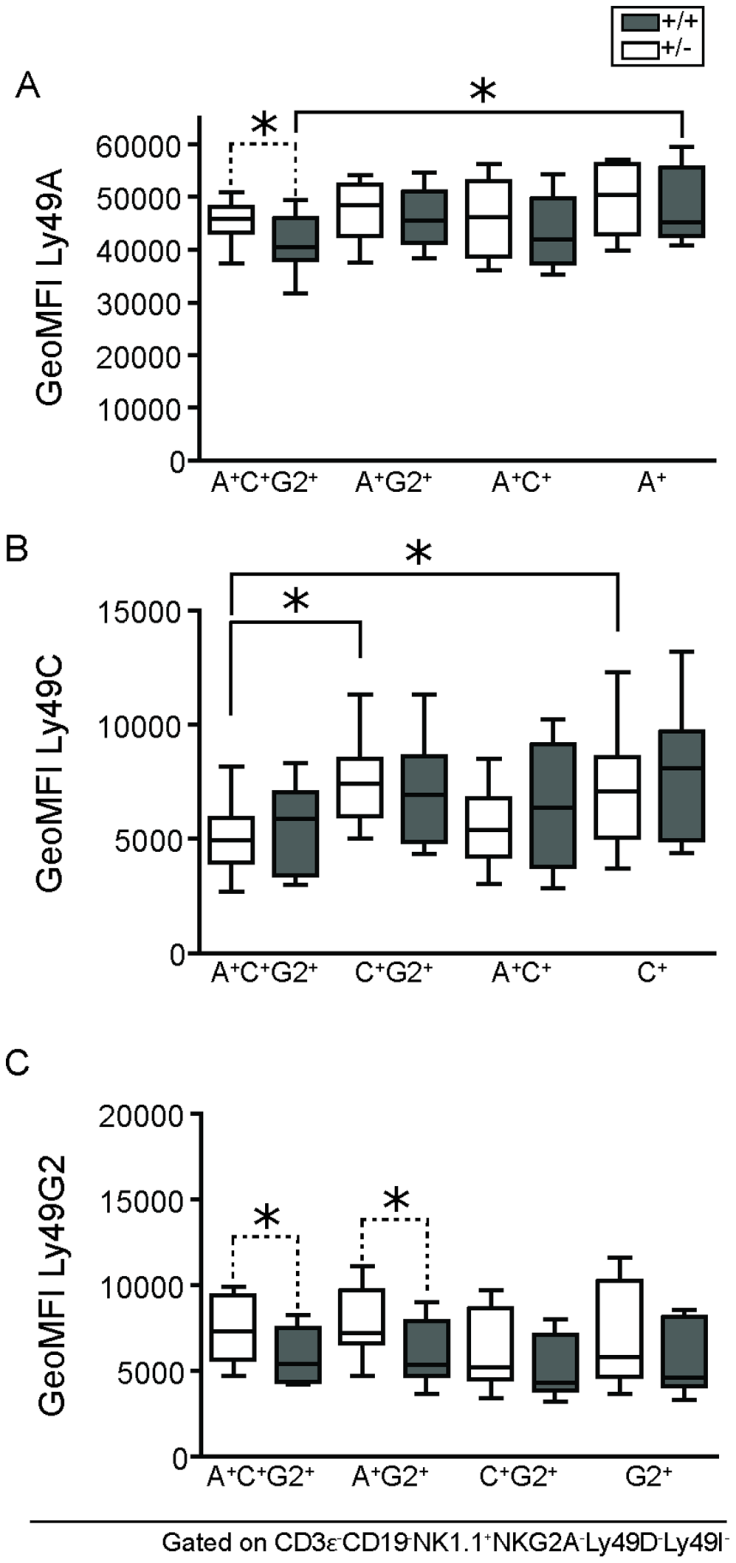
Expression levels of three D^d^-specific Ly49 receptors in NK cell subsets of hemi- and homozygous mice. Naive splenocytes from D^d+/+^ (grey boxes) and D^d+/−^ (white boxes) mice, stained *ex vivo* and gated on viable, CD3^−^CD19^−^NK1.1^+^ cells negative for NKG2A, Ly49D and Ly49I. Graphs show surface expression levels of A) Ly49A, B) Ly49C and C) Ly49G2 in the indicated subset combination. Data are combined from at least three independent experiments. Comparisons between subsets within strains were done using one-way ANOVA (solid lines) and between strains (dotted lines) using t-test. P-values: * <0,05.

Averages and standard deviations were calculated for measurements presented in Figures 2, 3, 4, 5, and 6, including the expected DMI values under the three above-described models. Significance of the differences between experimental groups and between the observed and expected values (in the modeling) was calculated using the standard Student's t-test. For comparisons between multiple populations in the experiment with Ly49 receptor levels on NK cell subsets, one-way ANOVA was used with Bonferroni's post test if p<0.05.

## Results

### MHC class I surface expression is sensitive to gene dosage

MHC class I expression is co-dominant, which means that both the paternal and maternal alleles are expressed. In accordance with this, we noticed in earlier experiments that the MHC class I surface expression level was lower in mice hemizyguous for a given MHC class I allele compared to mice carrying the same allele in a homozygous fashion. To investigate this phenomenon more systematically, and to quantify it for the K^b^ and D^b^ alleles, we crossed B6 mice with B6.K^b−/−^D^b−/−^ (MHC^−/−^) mice to generate mice hemizygous for both MHC alleles (see Table 1 for full genotypic names). Cell surface expression of K^b^ in K^b+/-^D^b+/−^ mice was reduced to 53±4.8% of the levels in K^b+/+^D^b+/+^ mice (Figure 1). The corresponding number for D^b^ was 71±10% (Figure 1).

To extend this analysis to an additional MHC class I allele, and to test whether this finding was also seen in mice expressing a single MHC class I allele, we crossed mice expressing either K^b^ or D^d^ as a single MHC class I allele on B6 background, with MHC^−/−^ mice, to generate K^b+/−^ and D^d+/−^ mice. In these mice, MHC class I levels were also reduced; in K^b+/−^ mice to 59% and in D^d+/−^ mice to 42% relative to homozygous controls (Figure 1). Thus, for three out of three analyzed MHC class I alleles, a gene dosage effect was observed for MHC class I expression, suggesting that MHC class I heavy chain transcripts represent a limiting factor in regulating MHC class I expression. A recent study found a similar effect also for a fourth allele, D^k^ [29].

### Target cells hemizygous for MHC class I molecules are not rejected by NK cells in MHC class I homozygous mice

We next tested the capability of K^b+/+^D^b+/+^ (wild-type B6) mice to react to the decreased expression levels of MHC class I on target cells from K^b+/−^D^b+/−^ mice. We used the previously described method for rejection of CFSE-labeled cells and injected hemizyguous K^b+/−^D^b+/−^ target cells into homozyguous K^b+/+^D^b+/+^ mice, together with syngeneic controls [6]. K^b+/+^D^b+/+^ mice were unable to reject K^b+/−^D^b+/−^ cells to any measurable extent (Figure 2A). This was seen over a time course of four days, indicating that it was not due to a delayed rejection, but rather to tolerance to these cells. In contrast, grafted cells completely lacking MHC class I molecules were rapidly rejected (Figure 2A). A similar result was seen for K^b+/−^ and D^d+/−^ target cells injected into K^b+/+^ and D^d+/+^ mice respectively (Figure 2B and C).

We also tested the overnight rejection of K^b+/−^D^b+/−^ cells in K^b+/+^D^b+/+^ mice pre-activated with the interferon-inducer tilorone. This pre-treatment leads to a general increase in NK responsiveness, presumably via mechanisms similar to those operating in viral infection [36]. Even in this experiment, K^b+/−^D^b+/−^ targets were tolerated (Figure 2D). Thus, both resting and tilorone-activated NK cells were insensitive to primary target cells with a down-regulation of surface MHC class I by 42–59%, providing strong evidence that a reduction in MHC class I expression of this magnitude is not enough to break NK tolerance to self, even if NK cells are activated by external stimulation.

### In vivo NK cell rejection requires loss of more than 80% of endogenuos MHC class I expression on target cells

We were surprised by the lack of rejection of normal cells despite loss of up to 60% of self MHC class I molecules at the cell surface. Since MHC class I-deficient cells are efficiently rejected, we hypothesized that there must be a threshold of MHC class I expression needed for protection against NK cell killing. This threshold would lie below approximately 40% of normal levels. To try to identify this threshold, we made use of Tap^−/−^ mice. These mice express low levels of peptide-empty MHC class I molecules, which can however be stabilized at the cell surface by the addition of exogenuous peptide [37]. We used a peptide (Mulv) derived from the Moloney murine leukemia virus, which up-regulates K^b^, but not D^b^, on B6.Tap^−/−^ cells [35], [38] (Figure S2B, time zero). Overnight incubation of B6.Tap^−/−^ splenocytes with 0.2–10 µM Mulv peptide induced K^b^ cell surface expression of 10 to 80% levels as compared to K^b+/+^ cells. The induced K^b^ level varied with the peptide concentration used, but also varied between experiments with the same peptide concentration.

We next examined *in vivo* rejection of B6.Tap^−/−^ cells with a stabilized K^b^ expression in K^b+/+^ mice (Figure 3). After 8 hrs, rejection of Mulv-stabilized B6.Tap^−/−^ cells was only seen when the K^b^ induction was below 20% of the level in K^b+/+^ mice (Figure 3). When K^b^ was induced above that level, the cells were protected from rejection, in most cases. The instability of the peptide-pulsed MHC class I complexes *in vivo* (Figure S2) prevented us from measuring the tolerance over a longer time; when the rejection assay was prolonged to overnight, peptide-induced K^b^ levels returned to Tap^−/−^ background levels and killing of peptide-stabilized cells also became apparent (data not shown). A longer observation time would have been useful to allow for better resolution between rejected and protected target cells. However, even if rejection of unpulsed cells was relatively small at 8 hours after injection, it was significant relative to NK1.1-treated controls (Figure 3). It should also be noted that the MHC class I level dropped during the time course of the experiment. Thus, the actual threshold could be even lower than the observed 20% of MHC class I induction, which would not be possible to control with this experimental setup. We conclude from this set of experiments that a surprisingly low number of self MHC class I molecules, approximately 20% of wild type, is sufficient to protect normal nontransformed cells from missing-self rejection *in vivo*.

### Ly49 receptor levels are unaffected by the reduced MHC class I levels in hemizygous mice

The surface expression level of Ly49 receptors are downmodulated by MHC class I through a combination of receptor internalization and *cis*-interactions [21], [22]. To test how a decrease in MHC class I levels would affect the degree of Ly49 receptor downmodulation, we measured Ly49A and Ly49C cell surface expression on splenic NK cells from MHC homozygous and hemizygous mice. Ly49A levels did not differ between K^b+/−^D^b+/−^ and K^b+/+^D^b+/+^ mice and were only marginally, if at all, lower when compared to MHC^−/−^ mice (Figure 4A). Ly49C was strongly downmodulated in both K^b+/−^D^b+/−^ and K^b+/+^D^b+/+^ mice when compared to MHC^−/−^ mice, but no significant difference was seen between K^b+/−^D^b+/−^ and K^b+/+^D^b+/+^ mice (Figure 4A). In the analysis of K^b^-single and D^d^-single mice, a similar pattern was observed with only minor differences between mice hemizygous and homozygous for the MHC allele (Figure 4B, C). Altogether, reduced MHC class I expression in hemizygous K^b^D^b^, K^b^ and D^d^ mice did not affect downmodulation of Ly49 receptors on NK cells, suggesting that MHC class I molecules are present in excess of Ly49 receptors at the cell surface of NK cells, at least when it comes to the mechanisms that induce Ly49 receptor downmodulation.

### Ly49 downmodulation is unaffected by the co-expression of additional low affinity MHC class I ligands

To further test the idea that MHC class I ligands might be in excess relative to Ly49 receptors, we examined how Ly49 receptor levels were influenced by the presence of several MHC class I alleles, each with a separate downmodulating capability on the same Ly49 receptor. To this end, we fitted observed downmodulation data to what would be expected by three different theoretical models, as follows: 1) The addition of several downmodulating MHC class I ligands would provide an additive effect on Ly49 downmodulation, so that the Ly49 receptor would be more downmodulated in the presence of multiple, compared to one single MHC class I ligand. 2) The most downmodulating MHC class I ligand would dominate, and the addition of weaker MHC class I ligands would have no additional effect on the Ly49 receptor expression level. 3) A stochastic competition exists between MHC class I molecules that would affect Ly49 downmodulation, such that the total downmodulation would be an average of the Ly49 downmodulating effects of individual MHC class I molecules.

To test these possibilities, we used mice that express different combinations of two or three MHC class I alleles [16]. Mice expressing at least two MHC class I molecules, that individually downmodulated the same Ly49 receptor, were chosen. To define when an MHC class I molecule had a downmodulating effect, we used the published statistically significant effects on Ly49 downmodulation in single MHC class I mice [16]. The MHC class I molecules that had a downmodulating effect in this study were D^b^, D^d^, and L^d^ for Ly49A, as well as K^b^, D^b^, and D^d^ for Ly49C. In Figure 5 (observed values), we show downmodulation of Ly49A and Ly49C in mice expressing combinations of these MHC molecules, as Down-modulation Indices (DMI). DMI of 0 means no down-modulation and 100 is complete down-modulation of the receptor. Next, we calculated the expected values under each of the theoretical models delineated above (dominant, additive, or averaged down-modulation, see Material and Methods for calculations).

The dominant model most frequently fitted best to the observed DMI (Figure 5). The average model could be statistically excluded for Ly49C (p<0.05 in all cases), but not for Ly49A. The additive model could not be statistically excluded for either receptor, possibly due to the high standard deviations calculated for this model. Yet, the overall pattern clearly suggested that a strongly downmodulating MHC class I molecule dominated the effect on Ly49 expression over any additional MHC class I molecules. Hence, MHC class I molecules with a weaker downmodulating effect lost this effect completely if co-expressed with a stronger MHC class I ligand. This supports our dominance model above and suggests that MHC class I molecules are indeed expressed in excess in relation to the Ly49 receptors.

### Coexpression of multiple Ly49 receptors on the same NK cell does not lead to increased cell surface expression of individual receptors

Downmodulation of Ly49 receptors by MHC class I can be used to predict which MHC class I alleles bind which Ly49 receptors [16]. Using this readout, we have previously identified D^d^ as a ligand for all three receptors Ly49A, Ly49G2 and Ly49C [16]. This notion is supported by previous MHC class I tetramer binding data from others [39] and also fits with the ability of D^d^ to functionally educate NK cell subsets expressing either one of these three receptors in isolation [40]. Given that Ly49A, Ly49G2 and Ly49C are all downmodulated by D^d^, a model in which MHC class I is limiting for Ly49 receptor downmodulation would predict that the cell surface expression level of at least one of those Ly49 receptors would be higher on NK cell subsets co-expressing these receptors, as compared to subsets expressing only one of the receptors. In contrast, if MHC class I molecules are present in excess, no such effect would be seen, and all of these Ly49 receptors would remain the same in NK cells expressing one, two or three of these receptors.

To test this prediction, we used a polychromatic flow cytometric protocol to measure the geometric Mean Fluorescence Intensity, geoMFI of Ly49A, Ly49C and Ly49G2 in all subsets expressing these receptors. To minimize contributions from other inhibitory receptors, NK cells expressing the receptors NKG2A and Ly49I were excluded. In addition, we also excluded the activating receptor Ly49D from the analysis, since this receptor binds D^d^ and could therefore affect the result. The same analysis including Ly49D^+^ NK cells, however, revealed only minor changes in the receptor levels, and the overall pattern remained (data not shown).

Our results show that none of the receptors Ly49A, Ly49G2 or Ly49C were expressed to a higher extent on NK cell subsets co-expressing multiple as compared to single Ly49 subsets (Figure 6, compare boxes with same color). This finding is in concordance with the hypothesis that MHC class I is expressed in excess of Ly49 receptors. In fact, somewhat surprisingly, the expression of Ly49A and Ly49C was in some cases even lower when coexpressed with Ly49G2, than when expressed in isolation (Figure 6A and 6B). We did note, however, higher expression of both Ly49A and Ly49G2 in D^d+/−^ *versus* D^d+/+^ mice for some NK cell subsets coexpressing one or two additional Ly49 receptors (Figure 6A and C). Thus, while data within each strain support a saturation effect of MHC class I on Ly49 receptor downmodulation, the comparison between hemizygous and homozygous mice suggest that MHC class I expression can be a limiting factor for receptor downmodulation on cells expressing multiple Ly49 receptors.

## Discussion

In this study, we addressed the influence of MHC class I levels on NK cell rejection of target cells, and on expression levels of Ly49 receptors. We found that cells expressing only 42% of the MHC class I level of the host on average are stably tolerated by NK cells *in vivo* over as long as four days, suggesting a robust tolerance over a range of MHC class I expression levels. It should be noted that the CFSE-based rejection method used in this study gives a quantitative measurement of rejection. Therefore, it is unlikely that we would have failed to detect rejection, even if only a small subset of the target population was killed.

Since tolerance to missing-self rejection was robust over a range of MHC class I expression levels, our data suggest a threshold effect for MHC class I on NK cell killing. A threshold effect has previously been suggested in an experiment with human NK cell clones and transformed targets [27]. It is also in line with the recently presented results by Jonsson et al, showing that *in vitro* killing is efficiently inhibited by ligands displaying moderate affinity to Ly49A, and that killing by D^d^ homozygote NK cells expressing Ly49A can be effectively inhibited by target cells expressing D^d^ hemizygously [29]. Such a threshold response in the effector stage could however be surprising given the recently published quantitative effect induced by the expression of MHC-Ly49 allelic pairs having different interaction strengths [16], [17], [18]. It was also not expected from earlier experiments suggesting that Ly49 receptor downmodulation would act as a mechanism to increase sensitivity to very small changes in MHC class I levels [41]. However, there could be physiological relevance (and thereby an evolutionary advantage) with a threshold, rather than a directly quantitative correlation, between MHC class I levels and inhibition. It is likely that the normal expression level of MHC class I molecules differs slightly between different cell types, or between different organs. Robust tolerance to such physiological differences in MHC class I levels would be a safeguard against triggering inadvertent autoimmune responses in the absence of disease.

In fact, the lack of reaction even by tilorone-activated NK cells emphasizes the idea of robust tolerance over a range of MHC levels. This result suggests that external activation of NK cells is not enough to break tolerance, and hence that further alterations of the target cell itself are required to trigger NK cell killing. These alterations could include upregulation of activating ligands, downregulation of alternative inhibitory pathways [42], [43], or further downregulation of MHC class I. These alternatives are not mutually exclusive; they could also work together to define what is a target cell compared to normal cells.

We also measured the effects of MHC class I on Ly49 expression levels and obtained data in support of a saturation model. First, our modeling showed that the effects of individual MHC class I alleles on Ly49 receptor downmodulation were not cumulative when several MHC class I alleles were coexpressed. The reason for this was that a strongly modulating allele already led to maximal downmodulation when expressed in isolation. Secondly, Ly49 receptors were not expressed at higher levels on NK cells expressing only one MHC allele, but several Ly49 receptors, on the same cell. This suggests that there was enough MHC class I available to allow full downmodulation of all Ly49 receptors at the same time. Only when MHC class I expression was limiting and several Ly49 receptors present at the same time, such as on NK cells in D^d^ hemizyguous mice, did we observe incomplete downmodulation of some Ly49 receptors. Altogether, the results support the saturation model, but also suggest the existence of a limit to this saturation.

An unexpected finding was that the Ly49C receptor, and to some extent also Ly49A, was expressed at even lower levels on cells coexpressing Ly49A, Ly49C and Ly49G2 in D^d^ mice. This could be a sign that additional mechanisms controlling Ly49 surface levels are at play, apart from downmodulation. We do not know at this stage if this would be an MHC-independent or MHC-dependent effect. MHC-independent mechanisms may exist that restrict the total number of Ly49 receptors at the NK cell surface. One reason could for example be competition for available space between Ly49 receptors in appropriate parts of the NK cell membrane. Alternatively, it could also be an MHC-dependent effect to limit the total extent of inhibition an NK cell receives, something that has been proposed to account for the lower frequency of NK cells with several self-specific Ly49 receptors [44]. The mechanisms could in either case be either post-transcriptional, leading to less Ly49 receptors reaching the cell membrane, or increased internalization of some Ly49 receptors. This is an intriguing possibility that would require further investigations to be clarified.

MHC class I interactions with Ly49 receptors have three measurable effects: Inhibition of NK cell activation, downmodulation of Ly49 expression levels, and education of NK cells to learn what is “self” MHC class I. These three effects could in turn be mediated via two types of Ly49-MHC class I interactions: *cis* or *trans*. In the case of inhibition of killing, it is obvious that the interactions take place through *trans* [27]. For the other two, the situation is less clear. It is known that Ly49 downmodulation to a large extent is mediated through *cis* interactions [19], [20], [45], but *trans* interactions could also play a role, at least when the NK cells themselves do not express any MHC ligand [21], [23], [46], [47], [48]. The role for *trans* and *cis* interactions in NK cell education is also unclear. Chalifour *et al* suggested that *cis* interactions may be required for NK cell education [20]. Most other models for NK cell education have assumed a dominant role of *trans* interactions, but the question is not very well addressed [10], [11], [17], [23], [49]. Acquisition of external MHC class I ligands from surrounding cells, and subsequent internalization in the NK cells' own membranes, also blurs the borders between *cis* and *trans* interactions [22], [50], [51]. MHC class I molecules originating from external cells could thus theoretically mediate effects through *cis* interactions on the NK cell itself.

Since inhibition of rejection and receptor downmodulation may, at least partly, be mediated through different types of MHC-Ly49 interactions (*cis* and *trans*), our data should not automatically be interpreted as if the threshold effect seen in rejection of hemizygous target cells, like receptor downmodulation, is also due to saturation. Even though *cis* and *trans* interactions take place using the same interaction sites on the Ly49 receptor, the avidity could be different in these two structurally different interaction processes, which would be supported by the reported differences in interaction mode [52]. This could in turn affect the threshold at which Ly49 receptors become saturated in the two different situations. Thereby, our data do not exclude the possibility that NK cells from homozygous mice actually do “sense” the lower levels of MHC class I on hemizygous cells, resulting in some type of altered intracellular signalling (affecting e. g. education, as discussed further below). However, because thresholds for activation are set higher, the NK cell still would not react with an effector action when confronted with hemizygous cells. It is thus not possible to conclude at this point whether or not tolerance to target cells expressing low levels of MHC class I is due to saturation of Ly49 receptors. However, Jonsson *et al* observed a correlation between the amount of *cis* interaction and inhibition of NK cell activation using MHC class I alleles having different affinities for Ly49A, which would support a connection between the amount of *cis* interaction and the degree of inhibition of activation [29]. This is an important issue, e. g. in attempts to manipulate NK cells to detect and react to putative target cells [53], and would therefore be an intriguing question to address in the future.

The third outcome of Ly49-MHC interactions, besides Ly49 downmodulation and inhibition of killing, is NK cell education and tuning. We did not address questions regarding education in this study. As for killing, precaution should be taken to interprete our results on “saturation” concerning receptor downmodulation, as also being valid for NK cell education. In addition to the differences between *cis* and *trans* interactions discussed above, education may also differ from receptor downmodulation and target cell killing in other aspects. For instance, educational interactions may take place at different times (during NK cell maturation) or in a different environment (displaying other MHC class I levels). Jonsson *et al* also studied education (or licensing). Their results would conform to the saturation effect [29]. However, there are also indications in the opposite direction. Studies in relation to the rheostat model, published by us [17], [18] and Joncker *et al* [54], indicate that MHC molecules may exert additive effects during education. Our ongoing studies also indicate that hemizygous MHC levels, may not be saturating in the educational process (manuscript in preparation). This would be in contrast to our finding that Ly49 downmodulation is saturated by the most strongly binding MHC class I, and shows that the degree of Ly49 downmodulation does not always correspond to the educating impact. Furthermore, saturation of Ly49 down-modulation in the presence of several MHC ligands was only supported by our data when the MHC class I allele was a strongly down-modulating one, as in the case of the K^b^-Ly49C and D^d^-Ly49A pairs. In the case of interactions leading to weaker down-modulations, such as D^d^ and D^b^ with Ly49G2, there was not such a clear-cut picture, and no statistically significant conclusions could be drawn (data not shown). The “threshold” in the saturating MHC class I level could thus potentially be different for different MHC-Ly49 pairs.

It should be noted that in the wild mouse population, due to the large number of alleles present at the MHC class I locus, the “normal” situation would most often be heterozygous expression of MHC class I. Quantitatively, this would correspond to “hemizygous” expression of each allele. It could therefore be that the NK cell system is not evolutionarily adapted to homozygous expression of MHC class I. Further work is needed in order to explore this interesting possibility. To what extent the “downmodulating effect” reflects the affinity of the MHC class I-Ly49 interaction is also not known. Clearly, surface plasmon resonance or similar measurements of the affinities of different Ly49-MHC class I pairs would be an advantage and bring progress to our understanding of the system.

To conclude, we have demonstrated in this study that NK cells are unable to sense a decrease by more than half of the MHC class I expression level on normal, resting cells, at least when it comes to Ly49 down-modulation as well as killing of target cells. In contrast to what was previously thought, mature NK cells are thus relatively insensitive to fluctuations in the expression levels of MHC class I molecules on normal cells. This may be a safeguard against aberrant reactions leading to autoimmunity, and may imply that other alterations of target cells, such as upregulation of activating ligands, or down-regulation of other redundant inhibitory ligands, may be crucial for the activation of NK cells.

## Supporting Information

Figure S1 Ly49 expression levels are not influenced by the MHC expression level in multiple MHC mice. The MHC class I expression level of the downmodulating allele in each case (x-axis) is taken from Table S1. The left panel shows Ly49C expression. The expression level of Ly49C (y-axis) is shown as a function of K^b^ expression level (x-axis) in K^b+/+^ and K^b^L^d+/−^ mice (squares), and as a function of D^b^ expression in D^b+/+^ and D^b^L^d+/−^ mice (triangles). MHC expression levels are shown as a fraction of K^b^ and D^b^ single MHC mice, respectively. The K^b+/+^ and D^b+/+^ mice are connected to the expression in MHC^−/−^ mice with a dotted line. The right panel shows Ly49A expression. The expression as a function of D^d^ expression level in D^d+/+^ and K^b^D^d+/−^ mice (circles) and the expression level as a function of D^b^ expression in D^b+/+^ and K^b^D^b+/+^ mice (triangles), as in A). The D^d+/+^ and D^b+/+^ mice are connected to the expression in MHC^−/−^ mice with a dotted line. If the Ly49 expression level were dependent on the MHC expression level, the hemizygous mice would be expected to fall on this line, since they only express approximately half of the MHC levels of the homozygous mice. However, this is in at least 3 out of 4 cases not the case. The exception is the expression of Ly49C as a function of D^b^ expression, which is a very weak interaction and therefore the amount of downmodulation is statistically uncertain.
(0.12 MB TIF)

Figure S2 Stability of MHC complexes at the cell surface after pulsing with Mulv peptide. A) In vivo stability of Mulv peptide. K^b^ single mice were treated with NK1.1 and injected with CFSE labeled B6.Tap^−/−^ cells pulsed with Mulv, along with K^b^ syngeneic controls. Their K^b^ levels were analyzed in blood after 2, 4, and 6 hours, and in the spleen after 6 hours. The reason for the increased expression of Kb on inoculated syngeneic cells during the experiment is unknown. B) Peptide binding to K^b^ and D^b^ and its stability tested in vitro. B6.Tap^−/−^ cells were pulsed with Mulv overnight at 26°C, washed and then put at 37°C for different time durations followed by staining and flow cytometry analysis of K^b^ and D^b^.
(0.14 MB TIF)

Table S1 MHC expression level in mice expressing two or three MHC class I alleles. MHC expression levels are given as a fraction of the expression in the respective single MHC mouse. In K^b^D^b^L^d^, K^b^D^b^D^d^, and K^b^D^b^ mice the K^b^ and D^b^ alleles were homozygously expressed. For all other alleles and mice the MHC was hemizygously expressed. Numbers in parenthesis indicate standard deviations.
(0.03 MB DOC)
