# Supplementary figures and images for: Natural Killer Cell Tolerance Persists Despite Significant Reduction of Self MHC Class I on Normal Target Cells in Mice
Source: PLoS One. 2010 Oct 4;5(10):e13174. doi: 10.1371/journal.pone.0013174 (PMC2949391; doi:10.1371/journal.pone.0013174)

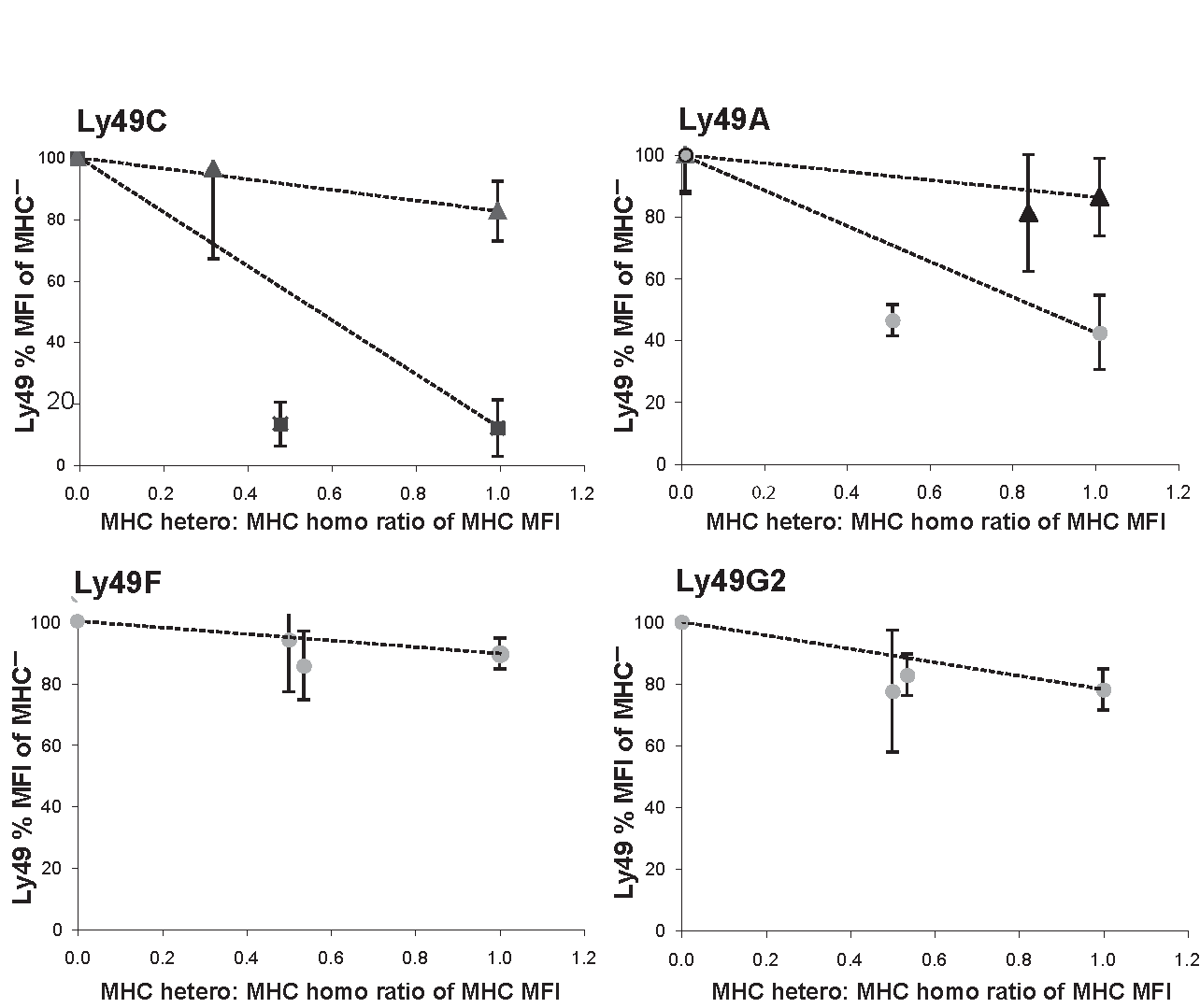

Supplement: Figure S1 — Ly49 expression levels are not influenced by the MHC expression level in multiple MHC mice. The MHC class I expression level of the downmodulating allele in each case (x-axis) is taken from Table S1. The left panel shows Ly49C expression. The expression level of Ly49C (y-axis) is shown as a function of Kb expression level (x-axis) in Kb+/+ and KbLd+/− mice (squares), and as a function of Db expression in Db+/+ and DbLd+/− mice (triangles). MHC expression levels are shown as a fraction of Kb and Db single MHC mice, respectively. The Kb+/+ and Db+/+ mice are connected to the expression in MHC−/− mice with a dotted line. The right panel shows Ly49A expression. The expression as a function of Dd expression level in Dd+/+ and KbDd+/− mice (circles) and the expression level as a function of Db expression in Db+/+ and KbDb+/+ mice (triangles), as in A). The Dd+/+ and Db+/+ mice are connected to the expression in MHC−/− mice with a dotted line. If the Ly49 expression level were dependent on the MHC expression level, the hemizygous mice would be expected to fall on this line, since they only express approximately half of the MHC levels of the homozygous mice. However, this is in at least 3 out of 4 cases not the case. The exception is the expression of Ly49C as a function of Db expression, which is a very weak interaction and therefore the amount of downmodulation is statistically uncertain. (0.12 MB TIF) [file pone.0013174.s001.tif]

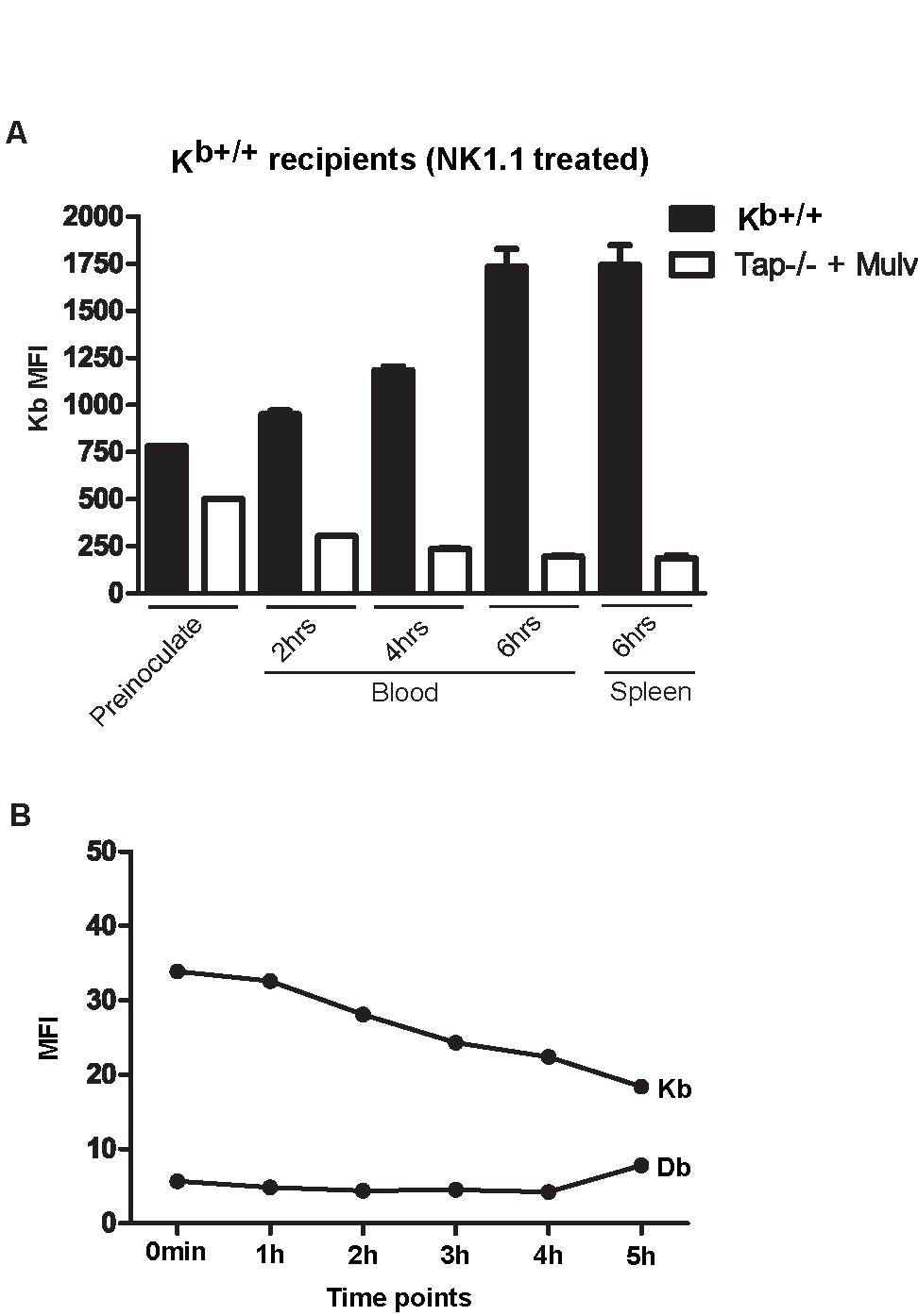

Supplement: Figure S2 — Stability of MHC complexes at the cell surface after pulsing with Mulv peptide. A) In vivo stability of Mulv peptide. Kb single mice were treated with NK1.1 and injected with CFSE labeled B6.Tap−/− cells pulsed with Mulv, along with Kb syngeneic controls. Their Kb levels were analyzed in blood after 2, 4, and 6 hours, and in the spleen after 6 hours. The reason for the increased expression of Kb on inoculated syngeneic cells during the experiment is unknown. B) Peptide binding to Kb and Db and its stability tested in vitro. B6.Tap−/− cells were pulsed with Mulv overnight at 26°C, washed and then put at 37°C for different time durations followed by staining and flow cytometry analysis of Kb and Db. (0.14 MB TIF) [file pone.0013174.s002.tif]
