# Supplementary material for: Natural Killer Cell Tolerance Persists Despite Significant Reduction of Self MHC Class I on Normal Target Cells in Mice
Source: PLoS One. 2010 Oct 4;5(10):e13174. doi: 10.1371/journal.pone.0013174 (PMC2949391; doi:10.1371/journal.pone.0013174)
Supplement: Table S1 — MHC expression level in mice expressing two or three MHC class I alleles. MHC expression levels are given as a fraction of the expression in the respective single MHC mouse. In KbDbLd, KbDbDd, and KbDb mice the Kb and Db alleles were homozygously expressed. For all other alleles and mice the MHC was hemizygously expressed. Numbers in parenthesis indicate standard deviations. (0.03 MB DOC) [file pone.0013174.s003.doc]

|  | **K^b^** | **D^b^** | **D^d^** | **L^d^** |
| --- | --- | --- | --- | --- |
| K^b^D^b^L^d^ | 0.92 (0.14) | 0.29 (0.04) |  | 0.95 (0.19) |
| K^b^D^b^D^d^ | 0.90 (0.12) | 0.67 (0.02) | 0.97 (0.12) |  |
| K^b^D^b^ | 1.04 (0.15) | 0.83 (0.07) |  |  |
| K^b^D^d^ | 0.58 (0.10) |  | 0.50 (0.08) |  |
| K^b^L^d^ | 0.48 (0.12) |  |  | 0.96 (0.04) |
| D^b^D^d^ |  | 0.52 (0.06) | 0.54 (0.10) |  |
| D^b^L^d^ |  | 0.32 (0.03) |  | 1.10 (0.11) |
